# Influence of Li_2_Sb Additions on Microstructure and Mechanical Properties of Al-20Mg_2_Si Alloy

**DOI:** 10.3390/ma9040243

**Published:** 2016-03-29

**Authors:** Hong-Chen Yu, Hui-Yuan Wang, Lei Chen, Min Zha, Cheng Wang, Chao Li, Qi-Chuan Jiang

**Affiliations:** Key Laboratory of Automobile Materials of Ministry of Education & School of Materials Science and Engineering, Nanling Campus, Jilin University, No. 5988 Renmin Street, Changchun 130025, China; hcyu14@mails.jlu.edu.cn (H.-C.Y.); DanLuo198709@gmai.com (L.C.); chengwang@jlu.edu.cn (C.W.); chaoli14hcyu14@mails.jlu.edu.cn (C.L.); jqc@jlu.edu.cn (Q.-C.J.)

**Keywords:** Al-Mg-Si alloy, mechanical properties, heterogeneous nucleation, primary Mg_2_Si

## Abstract

It is found that Li_2_Sb compound can act as the nucleus of primary Mg_2_Si during solidification, by which the particle size of primary Mg_2_Si decreased from ~300 to ~15–25 μm. Owing to the synergistic effect of the Li_2_Sb nucleus and adsorption-poisoning of Li atoms, the effect of complex modification of Li-Sb on primary Mg_2_Si was better than that of single modification of Li or Sb. When Li-Sb content increased from 0 to 0.2 and further to 0.5 wt.%, coarse dendrite changed to defective truncated octahedron and finally to perfect truncated octahedral shape. With the addition of Li and Sb, ultimate compression strength (UCS) of Al-20Mg_2_Si alloys increased from ~283 to ~341 MPa and the yield strength (YS) at 0.2% offset increased from ~112 to ~179 MPa while almost no change was seen in the uniform elongation. Our study offers a simple method to control the morphology and size of primary Mg_2_Si, which will inspire developing new Al-Mg-Si alloys with improved mechanical properties.

## 1. Introduction

The as-cast microstructure has a strong influence on mechanical properties of castings [1]. For Al-high Mg_2_Si alloy, the formation of primary Mg_2_Si reinforcement with small grain size and regular morphology is necessary to improve the mechanical properties of alloys and thus is has become the main issue when preparing the materials with excellent properties [2,3,4,5]. Intermetallic compound Mg_2_Si, which exhibits low density (1.99 × 10^3^ kg m^−^^3^), high melting temperature (1085 °C), high elastic modulus (120 GPa) and high hardness (4.5 × 10^9^ N m^−^^2^) as well as a low thermal expansion coefficient (TEC) (7.5 × 10^−^^6^ K^−^^1^), has been widely used as a reinforced phase to prepare Al/Mg_2_Si alloys [6,7,8,9]. The excellent properties of Mg_2_Si can make Al/Mg_2_Si alloys suitable for widespread use in automobile and aerospace fields [10,11,12,13]. However, under equilibrium solidification condition, primary Mg_2_Si tends to form coarse dendrite, which is harmful to the mechanical property of Al-Mg_2_Si alloys and limits their development and application [14,15,16]. Therefore, controlling the morphology and size of primary Mg_2_Si is a great challenge to material scientists [17].

As far as we know, modification treatment is the most effective method to control morphologies and sizes of primary and eutectic Mg_2_Si, which is readily available for commercial applications [18]. Among all kinds of modifiers, Sb has been widely used for modification treatment of primary and eutectic Mg_2_Si [19,20]. The reason is that Mg_3_Sb_2_ formed during solidification can act as the nucleus of primary and eutectic Mg_2_Si, refining the size of Mg_2_Si and improving mechanical properties of Al-Mg-Si alloys [19,20]. Alizadeh *et al.* [21] reported that with the addition of 0.2 wt.% Sb into the Mg-4Zn-2Si melt, flake-like eutectic Mg_2_Si changed into fine polygons, and the mechanical properties such as impression creep and hot hardness were improved significantly. In our previous study [22], we found that with the content of Sb addition increasing from 0 to 0.2 and to 0.5 and finally to 2 wt.%, the morphology of primary Mg_2_Si in Mg-4Si, alloys transformed from coarse dendrite to equiaxed-dendrite and to defective octahedron and finally to perfect octahedron; meanwhile, the morphology of eutectic Mg_2_Si transformed from flake-like to fine polygonal shapes. Based on the above research, one can see that the modification effect of Sb is more effective to eutectic Mg_2_Si than to primary Mg_2_Si. Therefore, how to enhance the modification effect of Sb on primary Mg_2_Si is the key to improving mechanical properties of Al-high Mg_2_Si alloy. However, only limited research has been reported regarding this issue.

Because the electronegativity difference between Li and Sb is relatively large, they could form compounds with thermodynamic stability such as Li_2_Sb and Li_3_Sb during solidification process. The calculated disregistry is 4.0% at the orientation relationship of ${\left(10\bar{1}0\right)}_{Li_{2}Sb}//{\left(111\right)}_{Mg_{2}Si}$ for Li_2_Sb while 5.8% at that of ${\left(001\right)}_{Li_{3}Sb}//{\left(001\right)}_{Mg_{2}Si}$ for Li_3_Sb, which are both less than 6.0% and may act as the nucleation substrate for primary Mg_2_Si [23]. To change morphologies and refine the size of primary Mg_2_Si during solidification and finally to improve the mechanical properties of Al-20Mg_2_Si alloy, we added Li and Sb simultaneously to the Al-Mg-Si melt. The mechanism of primary Mg_2_Si co-modified with Li and Sb was revealed in this research. The compression property and microhardness of Al-20Mg_2_Si alloys modified with 0, 0.2 and 0.5 wt.% Li-Sb were also tested. The results achieved will be a big step forward in realizing the artificial manipulation of grain refinement and morphology transformation of primary Mg_2_Si in Al alloys, which plays an important role in improving physical and mechanical properties of Al-Mg-Si alloys.

## 2. Experimental Section

### 2.1. Preparation of Al-20Mg_2_Si Alloy Modified with Various Contents of Li-Sb

In order to prepare Al-20Mg_2_Si alloy, where the unit of “20” is “wt.%” and the unit of “2” is the number of Mg atom in intermetallic compound Mg_2_Si, the contents of the Al ingot (99.98 wt.% purity), Mg ingot (99.85 wt.% purity) and Al-24.4Si master alloy are ~57.2 wt.%, ~12.6 wt.% and ~30 wt.%, respectively. The modifiers are pure Sb ingot (98.00 wt.% purity) and Mg-13.5Li master alloy. Pure Al and Al-24.4Si master alloy were melted at 750 °C in a graphite crucible in an electric resistance furnace of 5 kW; then pure Mg, Sb and Mg-13.5Li master alloy preheated at 150 °C in a vacuum oven were added to the melts together. The designed compositions of Li-Sb in melts were 0, 0.2 and 0.5 wt.%, with an atomic ratio of Li:Sb of 3:1. Manual agitation was conducted in the Al-Mg-Si melts for about 1 min and held at 750 °C for 20 min. Finally, the melts was poured into a steel mold preheated at 150 °C to produce Al-20Mg_2_Si alloy co-modified with various contents of Li and Sb.

### 2.2. Characterization

Metallographic samples with a size of 10 mm × 10 mm × 13 mm were cut at the bottom of the ingots. Metallographic samples were prepared by a standard procedure and etched with 0.5 vol.% HF-distilled water solution for about 30 s at room temperature. To observe the 3-D morphologies of primary Mg_2_Si, samples with the size of 1.2 mm × 12 mm × 13 mm were put into a 20 vol.% HNO_3_-distilled water solution to dissolve the Al covering on the surface of the primary Mg_2_Si. The samples for compression test were processed into cylinders of which the diameter is 3 mm and the height is 6 mm. X-ray diffraction (XRD) (D/Max 2500PC, Rigaku, Tokyo, Japan) was used to characterize phase constitutions of the samples, using CuK_α_ radiation in step modes from 20° to 80° with a scanning speed of 4° min^−1^ and an acquisition step of 0.02° (2θ). As-cast microstructures of Al-20Mg_2_Si alloy were investigated using optical microscopy (OM) (Carl Zeiss-Axio Imager A2m, Gottingen, Germany). The 3-D morphologies of the extracted primary Mg_2_Si were observed using a field emission scanning electron microscope (FESEM) (JEOL-6700F, JEOL, Tokyo, Japan). A scanning electron microscope (SEM) (EVO 18, Carl Zeiss, Mainz, Germany) equipped with an energy dispersive spectrometer analyzer (EDS) was used to observe the elemental surface scanning spectra. The nucleus of primary Mg_2_Si was explored by transmission electron microscopy (TEM) (JEM-2100, JEOL, Tokyo, Japan) equipped with an EDS analyzer (EDS6498, OXFORD, London, Britain) under an operating voltage of 200 kV. The compression tests of Al-20Mg_2_Si alloy were conducted in a MTS (INSTRON-5869, INSTRON, Boston, MA, USA) machine operating with a constant crosshead speed of height × 0.018 mm/min at room temperature. At least three compression tests were done for each condition to ensure the accuracy of results. The microhardness of Al matrix in Al-20Mg_2_Si alloy were tested by Microhardness Tester (1600-5122VD Microment 5104, Buehler, Chicago, IL, USA), and at least seven measurements were done for each condition to ensure the accuracy of the results.

## 3. Results and Discussion

### 3.1. Microstructure of Al-20Mg_2_Si Alloy Modified with Li and Sb Simultaneously

**Figure 1:**
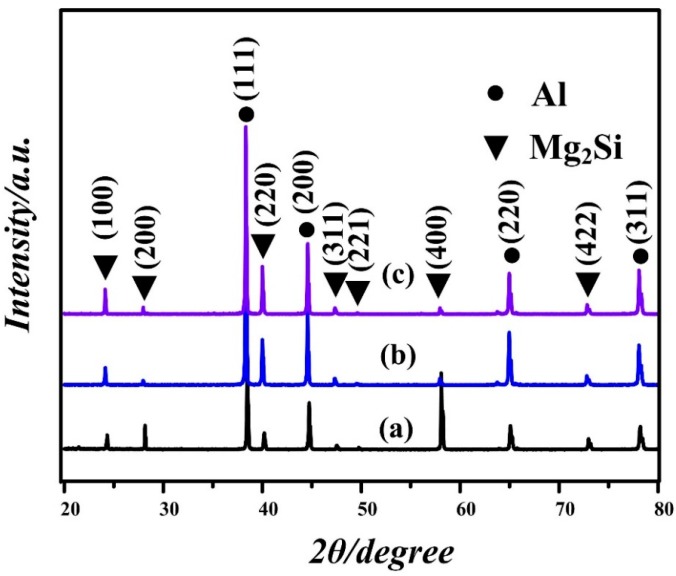
XRD patterns for Al-20Mg_2_Si alloy without and with various Li and Sb contents: (**a**) 0; (**b**) 0.2; and (**c**) 0.5 wt.% Li-Sb.

**Figure 2:**
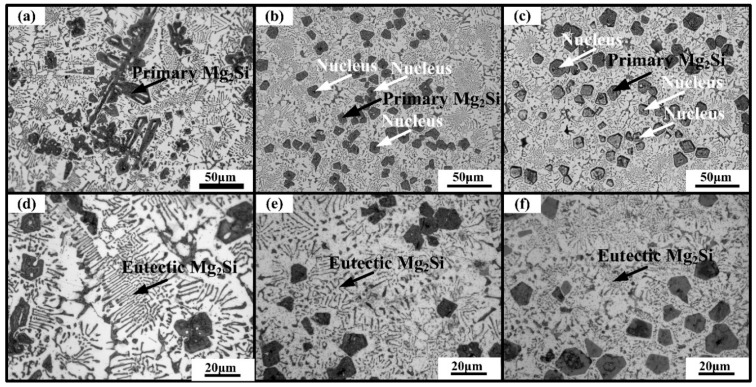
Microstructures of as-cast Al–20Mg_2_Si alloys without and with various Li-Sb contents: primary Mg_2_Si in (**a**) 0; (**b**) 0.2; and (**c**) 0.5 wt.% Li-Sb; eutectic Mg_2_Si in (**d**) 0; (**e**) 0.2; and (**f**) 0.5 wt.% Li-Sb.

According to the XRD results (Figure 1a–c), only Al and Mg_2_Si phases were found in the alloy. No characteristic peaks of compounds containing Li or Sb were detected in the modified alloys, which should be because the content of Li and Sb addition is limited. As-cast microstructures of Al-20Mg_2_Si alloys with 0, 0.2 and 0.5 wt.% Li-Sb additions are given in Figure 2a–f. With the addition of Li and Sb, the size of primary Mg_2_Si (see black arrows in Figure 2a–c) (Figure 2a) decreased from ~300 to ~15–25 μm and their morphologies changed into polyhedron (Figure 2b,c); the sizes of eutectic Mg_2_Si (see black arrows in Figure 2d–f) in modified alloys are also refined significantly despite the 2-D morphologies of eutectic Mg_2_Si still remaining flake-like (Figure 2d–f). Interestingly, one can see some dark spots occasionally located in the center of the polygons (see white arrows), which should be the nucleus of primary Mg_2_Si (Figure 2b,c).

**Figure 3:**
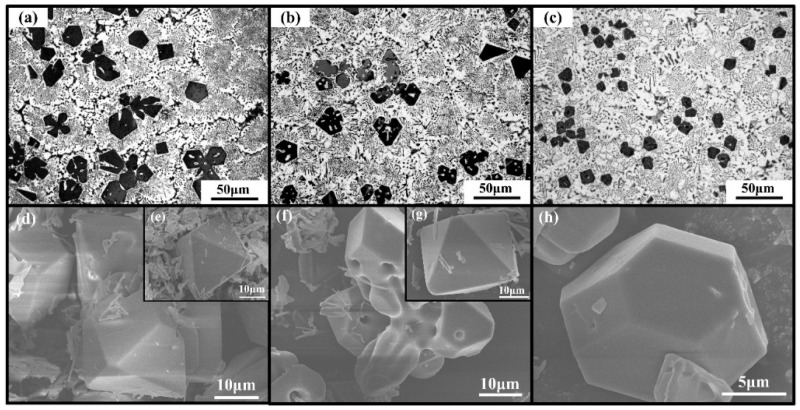
Microstructure images of primary Mg_2_Si in as-cast Al–20Mg_2_Si alloys modified with: (**a**) 0.2 wt.% Li; (**b**) 0.2 wt.% Sb; and (**c**) 0.2 wt.% Li-Sb. FESEM images of primary Mg_2_Si extracted from Al–20Mg_2_Si alloys modified with: (**d**–**e**) 0.2wt.% Li; (**f**–**g**) 0.2 wt.% Sb; and (**h**) 0.2 wt.% Li-Sb.

According to the literature [22,24], Li or Sb can restrict the growth of Mg_2_Si crystal by adsorbing on the growth sites of primary Mg_2_Si particles, and hence refine their size. For comparison, 0.2 wt.% Li and 0.2 wt.% Sb were separately added to Al-20Mg_2_Si alloys. As-cast microstructure of primary Mg_2_Si modified with 0.2 wt.% Li or Sb is shown in Figure 3a,b, respectively. Clearly, the grain refinement effect of 0.2 wt.% Li or Sb is relatively weaker than that of the combined addition of 0.2 wt.% Li-Sb (Figure 3c). Moreover, the 3-D morphologies of primary Mg_2_Si modified with 0.2 wt.% Li or Sb are also given (Figure 3d–g). As we can see, perfect octahedrons and equiaxed-dendrites were obtained in Al-20Mg_2_Si alloy modified with 0.2 wt.% Li (Figure 3d,e). Similar morphologies were also observed in the alloy modified with 0.2 wt.% Sb (Figure 3f,g). Meanwhile, truncated octahedral primary Mg_2_Si was formed when modified with 0.2 wt.% Li-Sb (Figure 3h). Apparently, compared with the modification effect of Li or Sb on primary Mg_2_Si, the co-modification effect of Li-Sb was enhanced significantly.

### 3.2. Characterization of Nucleus in Primary Mg_2_Si

**Figure 4:**
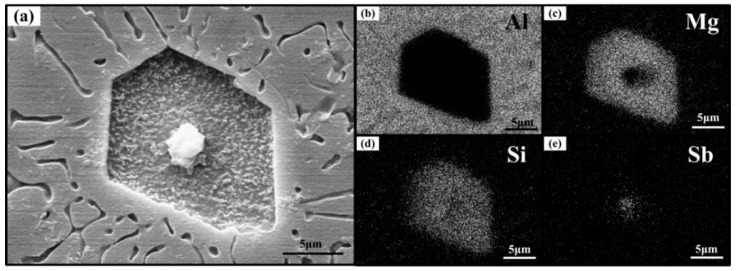
(**a**) The SEM micrograph and elemental surface scanning spectra for Al-20Mg_2_Si alloys modified with Li-Sb for: (**b**) Al; (**c**) Mg; (**d**) Si; and (**e**) Sb.

To identify the composition of the nucleus, shown in Figure 1b,c, elemental mapping scanning analysis was conducted. Note that the distribution of Li was not given because Li is a light element, which is difficult to be detected by EDS. As we can see, the Al atoms were mostly around the primary Mg_2_Si crystal (Figure 4b); Mg (Figure 4c) and Si (Figure 4d) atoms were detected in the crystal, while Sb atoms were mainly found inside the nucleus and the intensity of Sb (Figure 4e). Therefore, it is rational to say that the nucleus is a kind of antimony compound.

**Figure 5:**
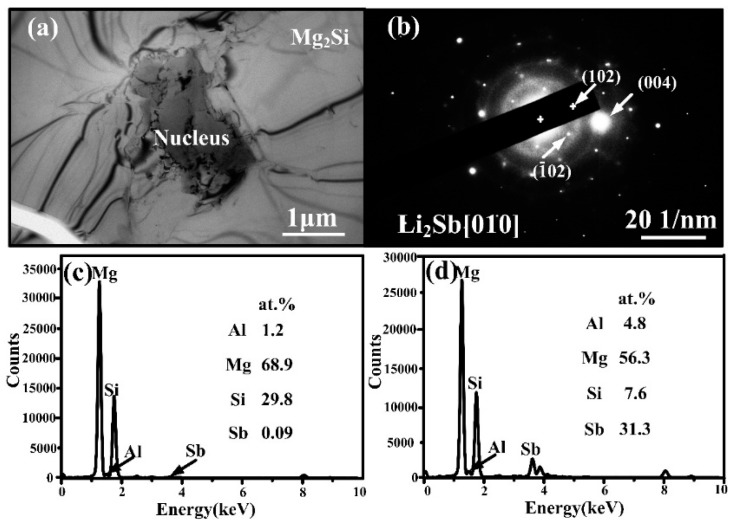
(**a**) TEM micrograph of the modified Mg_2_Si crystal with a nucleus; (**b**) selected-area diffraction (SAD) pattern of the nucleus in (a); (**c**) EDS for the modified Mg_2_Si crystal; and (**d**) EDS for the nucleus in (a), respectively.

Further investigation on the nature of nucleus was carried out by TEM and EDS. A nucleus located in the center of primary Mg_2_Si co-modified with Li-Sb is shown in Figure 5a. According to the double selected-area diffraction (SAD) pattern of nucleus (Figure 5b), the antimony-containing compound is Li_2_Sb, which has a hexagonal structure (P-62m) with the lattice constant of a = 0.7947 nm, b = 0.7947 nm, c = 0.3260 nm, α = β = 90° and γ = 120° [23]. In our previous study, we have confirmed that the Si sites in Mg_2_Si lattice can be substituted by Sb atoms when Sb was added into the Mg-4Si alloy [22], while no substitution occurred when Ca and Sb were simultaneously added to the Al-20Mg_2_Si alloy [22,25]. To investigate whether substitution occurred in the present case, the EDS analysis for the modified Mg_2_Si crystal and the nucleus is given in Figure 5c,d, respectively. According to the result, the EDS collected from the modified Mg_2_Si crystal contains mainly Mg, Si and Al peaks; only a few (0.09 at.%) Sb atoms were detected in the Mg_2_Si crystal (Figure 5c), while the EDS obtained from nucleus contains Mg, Sb (31.3 at.%), Si and Al peaks (Figure 5d). Thus, it can be concluded that most of the Sb atoms reacted with Li atoms to form Li_2_Sb compounds, acting as nucleus for Mg_2_Si crystals.

Note that, in our experiment, the designed atomic ratio of Li:Sb is 3:1, while the nucleus is Li_2_Sb, so that slight substitution of Sb atoms in Mg_2_Si lattice may also occur. In general, with the growth of crystal, the crystal facets with high growth rates will shrink gradually, while the facets with low growth rates will be reserved as crystal surfaces [26]. This suggests that some Li atoms did not react with Sb and they might be absorbed on the {100} facets. According to Figure 3a,d,e, sub-modification occurred in Al-20Mg_2_Si alloy with 0.2 wt.% Li added. Therefore, as for the primary Mg_2_Si modified with 0.2 wt.% Li-Sb, in addition to that Li_2_Sb nucleus can promote the nucleation of primary Mg_2_Si, additional Li atoms absorbed on {100} facets led to the exposure of {100} facets, and thus truncated octahedral primary Mg_2_Si formed, as shown in Figure 3h.

### 3.3. Effect of Li_2_Sb Nucleus on Mechanical Properties of Al-20Mg_2_Si Alloy

**Figure 6:**
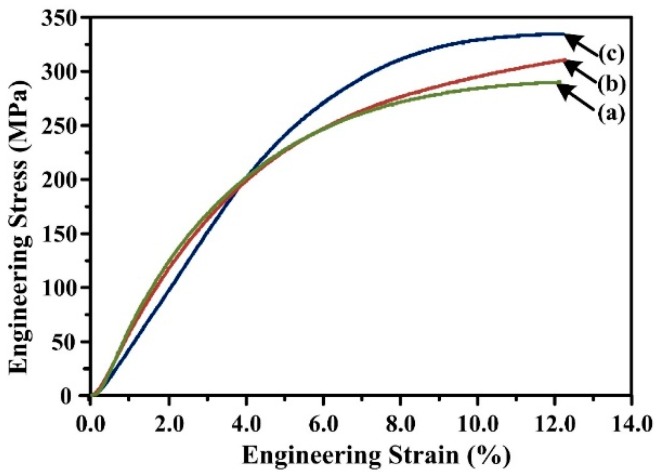
Engineering stress-stain curves of Al-20Mg_2_Si alloys: with: (**a**) 0; (**b**) 0.2; and (**c**) 0.5 wt.% Li-Sb addition.

The mechanical properties of Al-20Mg_2_Si alloys with 0, 0.2 and 0.5 wt.% Li-Sb addition are given in Figure 6 and Table 1. With the addition of Li and Sb, ultimate compression strength (UCS) of Al-20Mg_2_Si alloys increased from ~283 to ~341 MPa and the yield strength (YS) at 0.2% offset increased from ~112 to ~179 MPa, while almost no change was seen in the uniform elongation. The addition of Li and Sb also resulted in the increase in microhardness of α-Al matrix from ~91 to ~104 Hv. For a particle reinforced alloy, mechanical property is influenced by the reinforcement to a significant extent [10]. It is well known that primary Mg_2_Si is the reinforced phase in Al-20Mg_2_Si alloys and dendritic primary Mg_2_Si with a large size is harmful to mechanical properties [14,15,16]. Decreasing particle size usually leads to an increase in strength according to the Hall-Petch effect: [10].

$$\Delta \sigma_{YS}\approx D^{-1/2}{\left(\frac{V_{m}}{V_{\gamma }}\right)}^{1/6}$$

where Δ*σ_YS_* is the increment of yield strength; *D* is the size of reinforcement phase; and *V_m_* and *V_γ_* are the volume fraction of matrix and reinforcement, respectively. Thus, with the addition of Li-Sb, the size of primary Mg_2_Si decreases from ~300 to ~15–25 μm (Figure 2a–c), leading to improved UCS, YS, and microhardness of Al-20Mg_2_Si alloys.

However, it is worth to noting that with Li-Sb content increasing from 0.2 to 0.5 wt.%, similar microstructure features were observed and the size of primary Mg_2_Si still kept within the range of ~15–25 μm (Figure 2b,c), while the UCS increases significantly (from 306 to 341 MPa). Moreover, except Al and Mg_2_Si, no other phases that are beneficial to the mechanical properties of the alloy were detected (Figure 1a–c). Therefore, other factors, like the morphology of primary Mg_2_Si, may also influence mechanical properties of the Al-20Mg_2_Si alloy.

**Figure 7:**
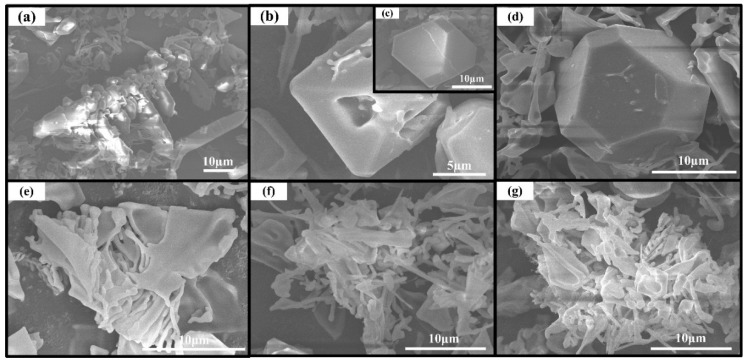
FESEM images of primary and eutectic Mg_2_Si extracted from Al–20Mg_2_Si alloys without and with various Li and Sb additions: primary Mg_2_Si in (**a**) 0; (**b**)–(**c**) 0.2; (**d**) 0.5 wt.% Li-Sb; and eutectic Mg_2_Si in (**e**) 0; (**f**) 0.2; and (**g**) 0.5 wt.% Li-Sb.

Typical 3-D morphologies of primary Mg_2_Si in Al-20Mg_2_Si alloys without and with various Li-Sb additions are given in Figure 7a–d. As we can see, with the content of Li and Sb increasing from 0 to 0.2 and then to 0.5 wt.%, the morphology of primary Mg_2_Si transformed from coarse dendrite (Figure 7a) to coexistence of defective truncated octahedron and perfect truncated octahedron (Figure 7b–c) and finally to a perfect truncated octahedral shape (Figure 7d). According to the literature, defective truncated octahedron can separate the α-Al matrix in the growth defect to some extent [3], leading to lower UCS of the alloy modified with 0.2 wt.% Li-Sb as compared to the alloy modified with 0.5 wt.% Li-Sb (Table 1). In addition, with the content of Li-Sb increasing from 0.2 to 0.5 wt.%, the size of eutectic Mg_2_Si decreased slightly (Figure 7e–g), which agrees well with the OM observations (Figure 2d–f). The refined size of eutectic phase is propitious to the improvement in the microhardness in modified alloys. Unfortunately, because Mg_2_Si particles are brittle, their existence is harmful to the plasticity of Al-20Mg_2_Si alloy [5,27]. Thus, controlling the morphology and size of primary Mg_2_Si has little effect on improving plasticity of the modified Al-20Mg_2_Si alloy.

## 4. Conclusions

In this paper, the effect of Li**_2_**Sb nucleus on microstructure and mechanical properties of Al-20Mg_2_Si alloys was investigated and the main conclusions are drawn as following:

(1) The 3-D morphology of primary Mg_2_Si was observed by extracting the Mg_2_Si crystals from Al–20Mg_2_Si alloys. With the addition of Li-Sb, the size of primary Mg_2_Si decreased from ~300 to ~15–25 μm and the morphology changed from coarse dendrite to defective truncated octahedron and finally to perfect truncated octahedral shape.
(2) The modification mechanism of Li-Sb can be concluded as follows: Li_2_Sb can act as better substrates to enhance the heterogeneous nucleation rate of primary Mg_2_Si; meanwhile, excess Li atoms were absorbed on and restricted the growth of {100} facets. The modification effect of Li-Sb was better than that of either Li or Sb, respectively.
(3) Influence of Li_2_Sb on mechanical properties of Al–20Mg_2_Si alloys was also investigated. With the addition of Li and Sb, ultimate compression strength (UCS) of Al-20Mg_2_Si alloys increased from ~283 to ~341 MPa and the yield strength (YS) at 0.2% offset increased from ~112 to ~179 MPa, while almost no change was seen in the uniform elongation. The addition of Li and Sb also led to the increase in microhardness of α-Al matrix from ~91 to ~104 Hv.

## Figures and Tables

**Table 1:** Mechanical properties of Al-20Mg_2_Si alloys modified with 0, 0.2 and 0.5 wt.% Li-Sb (the values following + signs were the upper limits while the value following – signs were the lower limits of the error bar).

Materials	YS/MPa	UCS/MPa	Uniform Elongation/%	Hardness/Hv
Al-20Mg_2_Si	$111.7_{-2.5}^{+2.6}$	$283_{-10}^{+8}$	$12.8_{-0.7}^{+1.0}$	$90.8_{-2.8}^{+2.0}$
Al-20Mg2Si-0.2(Li-Sb)	$121.7_{-3.1}^{+4.8}$	$306_{-6}^{+5}$	$12.1_{-0.6}^{+0.5}$	$100.4_{-3.1}^{+2.5}$
Al-20Mg2Si-0.5(Li-Sb)	$178.8_{-5.2}^{+3.1}$	$341_{-7}^{+9}$	$12.3_{-1.5}^{+1.3}$	$103.8_{-1.5}^{+1.1}$
